# Same Season and Carry-Over Effects of Source-Sink Adjustments on Grapevine Yields and Non-structural Carbohydrates

**DOI:** 10.3389/fpls.2021.695319

**Published:** 2021-07-26

**Authors:** Johann Martínez-Lüscher, Sahap Kaan Kurtural

**Affiliations:** Department of Viticulture and Enology University of California, Davis, Davis, CA, United States

**Keywords:** carbohydrates, carbon starvation, cluster thinning, crop load, defoliation, root growth, whole-plant physiology

## Abstract

The grapevine (*Vitis vinifera* L.) is managed to balance the ratio of leaf area (source) to fruit mass (sink). Over cropping in the grapevine may reveal itself as spontaneous fruit abortion, delayed ripening, or as alternate bearing. The aim of this work was to study the same season and carry-over effects of manipulating source to sink ratios on grapevine phenology, leaf gas exchange, yield components, berry soluble solids accumulation, and reserve carbohydrate and soluble sugar concentration in roots. Cabernet Sauvignon grapevines were subjected to defoliation (33, 66, and 100% of the leaves retained) and fruit removal treatments (33, 66, and 100% of clusters retained) arranged in a factorial design. Results from two seasons of source-sink manipulations were substantially different. In both seasons defoliation treatments affected season-long net carbon assimilation (*A*_*N*_) and stomatal conductance (*g*_*s*_) where the less leaves were retained, the greater the *A*_*N*_ and *g*_*s*_, and fruit removal had no impact on leaf gas exchange. In the first season, leaf area to fruit mass was hardly related to berry soluble solids and in the second season they were strongly correlated, suggesting a degree of acclimation. Defoliation treatments had great impacts on berry size, berries per cluster, and total soluble solids in both years. Fruit removal treatments only had effects on berry mass and berries per cluster in the first season, and only on berry soluble solids in the second. The predominant effect of defoliation (carbon starvation) cascaded onto reducing root starch content, root mass and delaying of veraison and leaf senescence, as well as harvest which was delayed up to 9 weeks with 33% of the leaves retained. In a third season, where grapevines grew without treatments, defoliation treatments had resultant carryover effects, including reduced leaf area, number of berries per cluster, clusters per vine, and yield, but not on leaf gas exchange dependent on previous seasons' severity of defoliation. Balancing source-to-sink ratio is crucial to obtain an adequate speed of ripening. However, this was the culmination of a more complex whole-plant regulation where the number of leaves (source strength) outweighed the effects of fruits (sink strength).

## Introduction

Grapevine (*Vitis vinifera* L.) has indeterminate growth habits compared to other perennial fruit crops. Latent growth of the dormant grapevine bud may be induced by favorable conditions with little to no dormancy period required (Williams, 2000; Martínez-Lüscher et al., 2016). Therefore, semi-tropical regions may raise two crops a year, and in fact, it is not uncommon for the latent bud to produce some fruit when correlative inhibition is removed in temperate regions. Furthermore, the grape berry does not have the same fruit abscission mechanism as apple (*Malus x domestica* Borkh.) or peach (*Prunus persica* L.) revealed under carbon starvation. It is therefore possible for grapevine canopy size and crop level manipulations leading to a wider range of source or sink limiting conditions within a growing season.

The crop level of a perennial crop is initially determined by organogenesis at the basal buds. The number and size of the flower primordia is associated with number of clusters and berries per cluster through the formation flowers and fruit set (Pool et al., 1978). However, fruit set is largely variable among years, weather, location, and cultivars (Keller, 2010). Poor fruit set may be a limitation to crop yield, although weather is often considered to be the leading cause. However, the mechanism of poor fruit set is not fully understood. Carbon supply or mineral nutrition are related to the amount of fruit set (Kliewer, 1977; Chaplin and Westwood, 1980; Caspari et al., 1998), which is an acclimation mechanism to unfavorable conditions. Ultimately, yield of grapevine is affected by berry size, and within the berry, pulp enlargement is the largest contributor to yield gain rather than skin or seed biomass (Walker et al., 2005). Conversely, vegetative growth is far less influenced by latent bud formation, as competition amongst growing buds tends to buffer the impact of growing shoot tips on its length and total leaf area (Greven et al., 2014). This is likely due to the great limiting effect of nitrogen among other nutrients or hydraulic pressure (Keller et al., 2015; Metay et al., 2015).

The ratio between leaf area and fruit mass is closely related to the amount of carbohydrates accumulated in the must (Naor et al., 2002). Thus, an excessive crop level or less than ideal canopy size may result in over cropping and may lead to delayed ripening (Geller and Kaan Kurtural, 2013). Conversely, in under cropping, where there is excessive vigor or reduced crop level, this is not necessarily deleterious for speed of ripening (Terry and Kurtural, 2011). However, it may be a wasteful management of resources if there is not a trade-off with farm-gate prices. Given the later fruit development of grapevine and the grape chemistry requirements for red wine making (Torres et al., 2020), the length of the growing season is often a limitation for achieving the desired ripening and vintage quality in cool climates (Jones and Davis, 2000). Thus, yield is often sacrificed to balance source-to-sink ratio in favor of accelerated fruit ripening or to mitigate the effects of early fall frosts (Terry and Kurtural, 2011; Gutiérrez-Gamboa et al., 2019). Although the initial control of crop level comes during pruning (Brandon O'Daniel et al., 2012; Wessner and Kurtural, 2013), the number of dormant buds retained at pruning time is maintained constant through the years in warm climate regions. Cluster thinning is a management practice fine-tuned each year to achieve vine balance (Kurtural et al., 2006; Terry and Kurtural, 2011; Wilson et al., 2014). Excess vine vigor was linked to deleterious effects on berry flavonoids (Baluja et al., 2012; Cook et al., 2015; Yu et al., 2016). This effect could be exacerbated with high nitrogen amounts inhibiting anthocyanin biosynthesis (Soubeyrand et al., 2014), the absence of water stress, or changes of cluster microclimate due to mutual shading (Keller et al., 2016; Brillante et al., 2018), and thus, not by the under cropping itself. Therefore, grapevine canopy development is managed through the control of inputs, vine spacing, irrigation, rootstocks, pruning, leaf removal, hedging, or cover crops, among others.

A great part of the carbon assimilated through the growing season is incorporated into cellulose or lignin in roots, trunks, and shoots (Greven et al., 2016). However, resumption of a new season's growth depends on the carbon stored as non-structural carbohydrates, majorly in the form of starch, but also soluble carbohydrates such as sucrose, glucose, and fructose (Greven et al., 2016). Roots are the greatest sink of non-structural carbohydrates and root-derived carbohydrates constitute the principal reserve source for annual resumption of growth in the spring. The grapevine's capacity for replenishment of these carbohydrate reserves increases at mid-ripening, when canopies are at their maximum and fruit demand slows down sugar accumulation in perennial parts (Candolfi-Vasconcelos et al., 1994). Therefore, the loss of photosynthetically active leaf area or excessive number of clusters may impair the reconstitution of reserves (Torres et al., 2021). In addition, high crop levels may delay fruit maturation and shorten the post-harvest period and subsequently reduce the time needed to accumulate reserve carbohydrates. Grape growing systems based on high yields are typically in warm to hot regions, relying on early harvest to replenish these reserves. However, it is common that excessive yields lead to a reduction in yields the following season (Geller and Kaan Kurtural, 2013; Kurtural et al., 2019). Loss of photosynthetically active leaf area or excessive number of clusters may deplete these reserves. High crop levels may reduce the reserve carbohydrate accumulation and delayed fruit maturation and may shorten the post-harvest period. Therefore, the grapevine may not have sufficient time to accumulate carbohydrates for the following season in cool climates. Conversely, there is not consensus in literature regarding the effect of high cropping levels on storage reserves (Bravdo et al., 1985). This was explained by sink limitation as the grapevine was able to maintain equilibrium by adjusting physiological processes (Poni et al., 2006; Kurtural et al., 2013).

In addition to the modulation of berry ripening and storage reserves, other compensatory mechanisms have been described in response to over and under cropping. Components of yield, which include clusters per vine, berries per cluster, and berry mass, are susceptible to change together with berry ripening in compensation of each other (Palliotti and Cartechini, 1998; Greven et al., 2014). Although grapevine pruning, canopy, and crop load management are the most frequently reported case of study for source-to-sink ratios, most studies may not offer direct observations (i.e., modulation of source-to-sink ratio given by background conditions), enough combinations, duration of the study, or range of source-to-sink ratios to respond to some fundamental questions. The aim of this study was to determine the in-season and carryover effects of carbon source and sink imbalances in grapevine. Specifically, we investigated the combined effects of defoliation and fruit removal on components of yield, canopy area, and seasonal integrals of leaf gas exchange, shifts in phenology, carbohydrate, and soluble sugar concentration in the roots.

## Materials and Methods

### Experimental Site and Plant Material

The experiment was conducted at the University of California Davis, Oakville Experimental Vineyard (38.428, −122.409; Oakville, CA) from 2017 to 2019 over three growing seasons. Eight-year-old *Vitis vinifera* “Cabernet Sauvignon” Clone FPS08 grafted on 110 Richter (*Vitis berlandieri x Vitis rupestris*) rootstock were used. Plants were trained to a bilateral cordon, manually pruned to 24 buds. The shoots were vertically shoot-positioned. Row and vine spacing was 2.4 × 2.0 m, respectively, and rows were oriented Northwest to Southeast. The plants were drip-irrigated with two pressure compensating emitters per plant delivering 2.0 L/h each. The plants were irrigated from fruit-set to end of harvest at 0.5 of crop evapotranspiration replacement as previously reported (Yu and Kurtural, 2020).

### Experimental Design and Treatment Application

**Figure 1:**
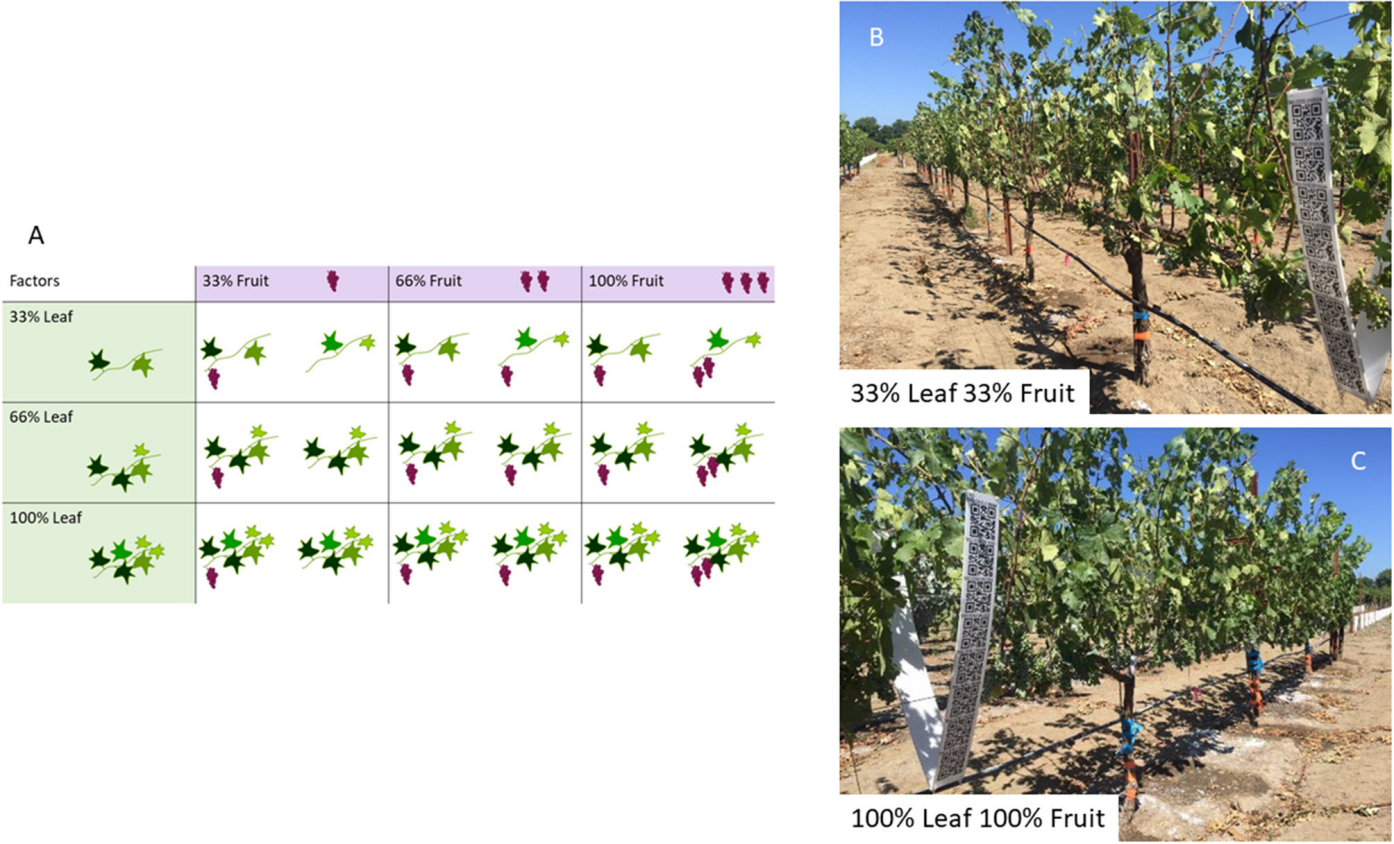
Amount of defoliation and crop removal conducted to achieve the treatment levels desired in the experiment. Amount of defoliation and fruit removal conducted to achieve the treatment levels desired in the experiment **(A)**, most extreme defoliation and fruit removal treatment **(B)** and least extreme defoliation and fruit removal treatment **(C)**.

The experimental design was a factorial arrangement of treatments. There were three levels of manual defoliation (retaining 100, 66, or 33% of the leaves) by three levels of manual fruit removal (retaining 100, 66, or 33% of the clusters) applied (Figure 1A). The treatments were applied as follows. Leaves were removed on every shoot in an alternating pattern. For instance, 66% of leaf treatments retained leaves in positions 1st, 2nd, 4th, 5th, 7th, 8th etc. while 33% of leaf treatments kept leaves in positions 1st, 4th, 7th, etc. in every shoot (Figures 1B,C). The fruit removal treatments retained a percentage of clusters (100, 66, and 33%) after standardizing the cluster numbers in each year. Each treatment combination was replicated four times (*n* = 36) and each treatment-replicate consisted of three experimental units. In 2017, all vines were standardized at fruit set to 20 shoots and 30 clusters per vine, and laterals were removed prior to defoliation and fruit removal treatments. In 2018, all vines were standardized to 24 shoots and 45 clusters and laterals were removed prior to treatment application. Treatments were applied at pepper-corn size (E-L number 29; Coombe, 1995). In 2019, after two seasons of growth under the nine combinations of treatments, the carryover effects were studied by leaving all vines untreated (i.e., no defoliation or fruit removal applied). For each experimental unit one vine was shoot thinned to 24 shoots, and others were left unmanaged (free vegetation). All clusters at pepper-corn size (E-L number 29) in all treatment-replicates were dipped in a 5.5% (v/v) kaolin solution to provide protection from the afternoon sun due to the row orientation of the vineyard in every year of the experiment.

### Primary Metabolism

#### Phenology

Percentage of bud break (E-L number 4), flowering (E-L number 23), veraison (E-L number 35), and leaf senescence (E-L 45) per plant were recorded at time intervals of either 1, 2, or 3 times a week depending on weather and phenology events (Coombe, 1995). A leaf was considered senescent when 50% of its area was yellow. Measurements started soon after the application of treatments in 2017 (pepper-corn size) until leaf senescence 2019. In 2019, only grapevines thinned to 24 shoots per vine were followed.

#### Leaf Gas Exchange

Leaf gas exchange was measured bi-weekly in all years of the experiment with an infra-red gas analyzer (CIRAS3, PP Systems, Amesbury, MA). Three sun-exposed leaves were selected from the main shoot axis in each experimental unit, and three readings were taken from each leaf. Gas exchange measurements were taken when the sunlight conditions were close to saturating levels in all instances. The relative humidity was set at 40%, the reference CO_2_ concentration was set at 400 μmol CO_2_ mol^−1^ as the standard environmental condition setting in CIRAS-3. Net carbon assimilation rate (*A*_N_, μmol m^−2^ s^−1^) and stomatal conductance (*g*_s_, mmol m^−2^ s^−1^) were obtained. To express the season-long response of *A*_*N*_, and *g*_s_, their integrals were calculated by using natural cubic splines for plant water status and gas exchange measurements to assess the cumulative values for these parameters over the whole experiment period during the growing season. Then, these cumulative values were normalized as divided by the number of days elapsed between the first measurement date and the last measurement date to make the data comparable to each individual measurement.

#### Leaf Area, Plant Mass, Components of Yield, and Must Soluble Solids

After harvest, leaves from one vine per replicate were collected, weighted, and dried in a forced-air oven at 80°C for 3 days. Dry leaf weights were converted into area by measuring the area of a subsample of 50 random leaves with a leaf area meter (LI-COR, 3100C, Lincoln, Nebraska) as reported previously (Yu and Kurtural, 2020).

On 12 December 2018, after the second season of treatments, one vine per experimental unit of the most extreme treatments (33% and 100% combinations) were pruned, coppiced, and the root systems were removed with a back-hoe. The sectioned grapevine portions (roots, trunk, shoots) were weighed on a top-loading scale, and dried in a forced-air oven at 60°C until no weight change of tissue was detected.

At harvest (~25°Brix for each treatment combination), clusters were removed, counted, and weighed for each plant in the experiment. Total soluble solids were measured from 55 berries collected randomly at harvest point. The berries were crushed by hand and filtered to obtain must. A digital refractometer (Palette PR-32, Atago, Tokyo, Japan) was then used to measure total soluble solids (TSS) of must.

#### Root Starch and Soluble Sugars

Soon after the harvest of 2017 was completed, root tissues were sampled every 2 months. The top layer of soil was removed (typically 0.3 m deep) until the roots were visible. Each grapevine root zone was divided into four quadrants and on each date and one single quadrant was sampled, leaving the other 11 quadrants undisturbed. Roots were gently cleaned with water, freeze-dried, and ground to a fine powder with a tissue lyser (MM400, Retsch, Germany). Thirty milligrams of the resultant powder were extracted in 80:20 ethanol solution. A 1.5 mL aliquot of the extract was then placed in a 90°C water bath for 10 min, then centrifuged at 10,000 rpm for 1 min. The supernatant was collected for total soluble sugars determination. The same procedure was repeated for starch determination, in which the pellet was collected for its determination.

Total soluble sugars in the roots were determined as reported elsewhere by Torres et al. (2020). Briefly, the 1.5 mL sample was filtered by PTFE membrane filters (diameter: 13 mm; 0.45 μm; Celltreat Scientific Products, Pepperell, MA, USA) and transferred into high performance liquid chromatography (HPLC) vials. Equipment consisted of a reversed-phase HPLC system Agilent 1100 coupled to a diode array detector (DAD) and an Agilent Infinity Refractive Index Detector (RID) (Agilent, Santa Clara, CA, USA). The reversed-phase column was Luna Omega Sugar (150 x 4.6 mm, 3 μm particle size) with a guard column of 5 mm. The temperature of the column compartment was maintained at 40°C and the RID flow cell was kept at 35°C. The mobile phase system consisted in an isocratic elution with acetonitrile:water (v:v, 75:25) at a flow rate of 1.0 mL•min^−1^ with a run time of 22 min. Standard solutions of 10 mg/L of D-glucose, D-fructose, D-sucrose, and D-raffinose were injected to obtain the retention time for each compound, and detection was conducted by RID. Sugar standards were purchased from VWR (Visalia, CA). Sugar concentration of each sample was determined by comparison of the peak area and retention time with standard sample curves.

Starch content of roots was measured using the Starch Assay Kit SA-20 (Sigma, St. Louis, MI, USA) following the manufacturer's instructions. Briefly, pellets of root tissues were dissolved in 1 mL DMSO, and incubated for 5 min in a water bath at 100°C. Starch digestion was started by adding 10 μL α-amylase and incubated in boiling water for another 5 min. then, the ddH2O was added to a total volume of 5 mL. Then, 500 μL of the above sample and 500 μL of starch assay reagent were mixed and incubated for 15 min at 60°C. Negative controls with the starch assay reagent blank, sample blank, and glucose assay reagent blank and positive controls with starch from wheat and corn were performed. Reaction started with the incubation of 500 μL of each sample and 1 ml of glucose assay reagent at 37°C and was stopped with the addition of 1 mL of 6 M Sulfuric acid after 30 min. Reaction was followed with analytical measurements with a Cary 100 Series UV-Vis Spectrophotometer (Agilent, Santa Clara, CA, USA) and starch content expressed as mg of starch per tissue dried weight.

### Statistical Analysis

The same grapevines were measured on each date throughout the execution of the experiment. Season-long measurements of root starch and soluble sugars, and phenology were analyzed separately for each year via three-way ANOVA for a date × defoliation × fruit removal design using PROC MIXED procedure of SAS (v 9.4. SAS Institute, Cary, NC) using REPEATED option for measurement dates. Measurements of season-long leaf gas exchange integrals, grapevine vegetative growth, yield and yield components, and total soluble solids variables were analyzed via three-way ANOVA for year × defoliation × fruit removal using the same procedure of SAS. Whenever the year and treatment interactions were significant the analyses was conducted by year. *Post-hoc* analyses were conducted using Tukey's HSD at *p* < 0.05. The trend analysis was carried to the quadratic level and was conducted with planned orthogonal contrasts using PROC GLM procedure of SAS. Certain variables (percent change in phenology) were log-transformed based on most-likelihood analysis.

## Results

### Effects of Source Sink Adjustments on Components of Yield

In our experiment the results indicated that there was an interaction of year and defoliation on cluster weight, berries per cluster and yield per vine (Table 1). When we analyzed the data by year, the effect of defoliation was clearer. In both experimental years (2017–2018), there was a strong linear trend of defoliation on all components of yield except for cluster number; which was only affected by the fruit removal treatments. In 2017 defoliating 66% of the leaves resulted in an 8% decrease in berry weight. The differences were exacerbated in cluster weight (32%), berries/cluster (25%), and yield (33%) when 66% of the leaf area was removed. In 2018, the effect of defoliation was evident with a 12% decrease in berry weight. As in the previous year, we saw a diminution in cluster weight, berries/cluster, and yield. However, the decline in yield in 2018 was 56% when 66% of the leaves were defoliated.

**Table 1:** Effects defoliation and fruit removal on components of yield on Cabernet Sauvignon/110R in two successive seasons (2017–2018).

**Factor**	**2017**				
**Leaves retained**	**Berry wt. (g)**	**Cluster no**.	**Cluster wt. (g)**	**Berries/cluster**	**Yield (kg)**
33%	0.79 b^a^	20	79.2 b	101.1 b	1.57 b
66%	0.86 a	20	103.5 b	120.6 a	2.05 a
100%	0.86 a	20	116.46	135.1 a	2.35 a
*P*	0.0150	0.5884	0.0001	0.0001	0.0001
**Trend**^**b**^					
Linear (P)	***	NS	***	***	***
Quadratic (P)	NS	NS	NS	NS	NS
**Fruit retained**					
33%	0.85	10 c	107.9 a	127	1.23 c
66%	0.84	20 b	99.9 ab	118	1.97 b
100%	0.81	30 a	91.4 a	112	2.78 a
*P*	0.3094	0.0001	0.0035	0.0637	0.0001
**Trend**					
Linear (P)	NS	***	*	NS	***
Quadratic(P)	NS	NS	NS	NS	NS
Leaves × Fruit (P)	0.5774	0.3135	0.8017	0.8137	0.1773
**Leaves retained**	**2018**				
33%	0.94 b	29	93.8 c	99 b	2.73 b
66%	1.02 ab	32	113.3 b	110 ab	3.59 a
100%	1.07 a	31	128.5 a	119 a	3.99 a
*P*	0.0021	0.1272	0.0001	0.0025	0.0001
**Trend**					
Linear (P)	***	NS	***	***	***
Quadratic (P)	NS	NS	NS	NS	NS
**Fruit retained**					
33%	0.99	17 c	115.3	116	1.91 c
66%	1.02	33 b	109.0	106	3.57 b
100%	1.02	44 a	111.3	108	4.83 a
*p*	0.6131	0.0001	0.5784	0.1243	0.0001
**Trend**					
Linear (P)	NS	***	NS	NS	***
Quadratic (P)	NS	*	NS	NS	NS
Leaves × Fruit (P)	0.7576	0.5487	0.7340	0.9230	0.0984

a *Means with a different letter indicate significant different at P < 0.05 according to Tukey's HSD (n = 36)*.

b Trend analysis conducted using single degree of freedom planned orthogonal contrasts and carried to the quadratic level. NS, *, and *** indicate non-significance or significance at P < 0.05 and P < 0.0001 probability levels, respectively.

Fruit removal was effective in modulating the cluster number and thus the yield in both experimental years as expected (Table 1). Furthermore, we measured a strong linear decrease in cluster weight in 2017. However, the same response was not evident in 2018. Removing 66% of the cluster resulted in a 55 and 60% decrease in yield of Cabernet Sauvignon in 2017 and 2018, respectively. Surprisingly, we did not measure an interaction of defoliation and fruit removal on components of year in either of the experimental years (Table 1).

The carry-over effects of source-sink adjustments on components of yield in 2019 were strongly evident (Table 2); even though no defoliation or fruit removal treatments were applied. Berry weight, cluster number per vine, cluster weight, and yield per vine were all affected by the carry over effects of defoliation from the previous 2 years. They all declined linearly with the 33% defoliation treatment. Conversely, we did not measure a carryover effect of fruit removal in 2019 in the majority of components of yield monitored. There was an interaction of defoliation and fruit removal in 2019 on the number of berries per cluster. The 33%L-100%F had the fewest berries per cluster compared to all other treatment combinations.

**Table 2:** Carry-over effects of defoliation and fruit removal on components of yield on Cabernet Sauvignon/110R after two successive seasons.

**Factor**	**2019**				
**Leaves retained**	**Berry wt. (g)**	**Cluster no**.	**Cluster wt. (g)**	**Berries/cluster**	**Yield (kg)**
33%	0.95 b^a^	86 b	57.5 b	105 a	4.89 c
66%	1.01 ab	101 ab	65.1 ab	95 b	6.59 b
100%	1.04 a	116 a	68.1.a	105 a	7.89 a
*P*	0.0218	0.0041	0.0071	0.0019	0.0001
**Trend**^**b**^					
Linear (P)	***	***	***	***	***
Quadratic (P)	NS	NS	NS	NS	NS
P					
**Fruit retained**					
33%	0.97	99	65.3	97	6.41
66%	0.99	100	61.4	98	6.18
100%	1.03	104	64.1	94	6.78
*p*	0.2157	0.7812	0.4545	0.7572	0.4285
**Trend**					
Linear (P)	NS	NS	NS	NS	NS
Quadratic (P)	NS	NS	NS	NS	NS
Leaves × fruit (P)	0.3827	0.4198	0.8515	0.0101	0.2998
Year	0.0001	0.0001	0.0001	0.0001	0.0001
Year × leaves	0.6611	0.4142	0.0008	0.0001	0.0002
Year × fruit	0.2367	0.0004	0.1363	0.2795	0.0001
Year × fruit × leaves	0.9431	0.5682	0.7861	0.2960	0.8709

a *Means with a different letter indicate significant different at according to Tukey's HSD (n = 36)*.

b Trend analysis conducted using single degree of freedom planned orthogonal contrasts and carried to the quadratic level. NS and *** indicate non-significance or significance at P < 0.0001 probability level, respectively.

### Source Sink Adjustments on Canopy Area, Leaf Area to Fruit Ratio, Ravaz Index, and Berry Ripening

**Figure 2:**
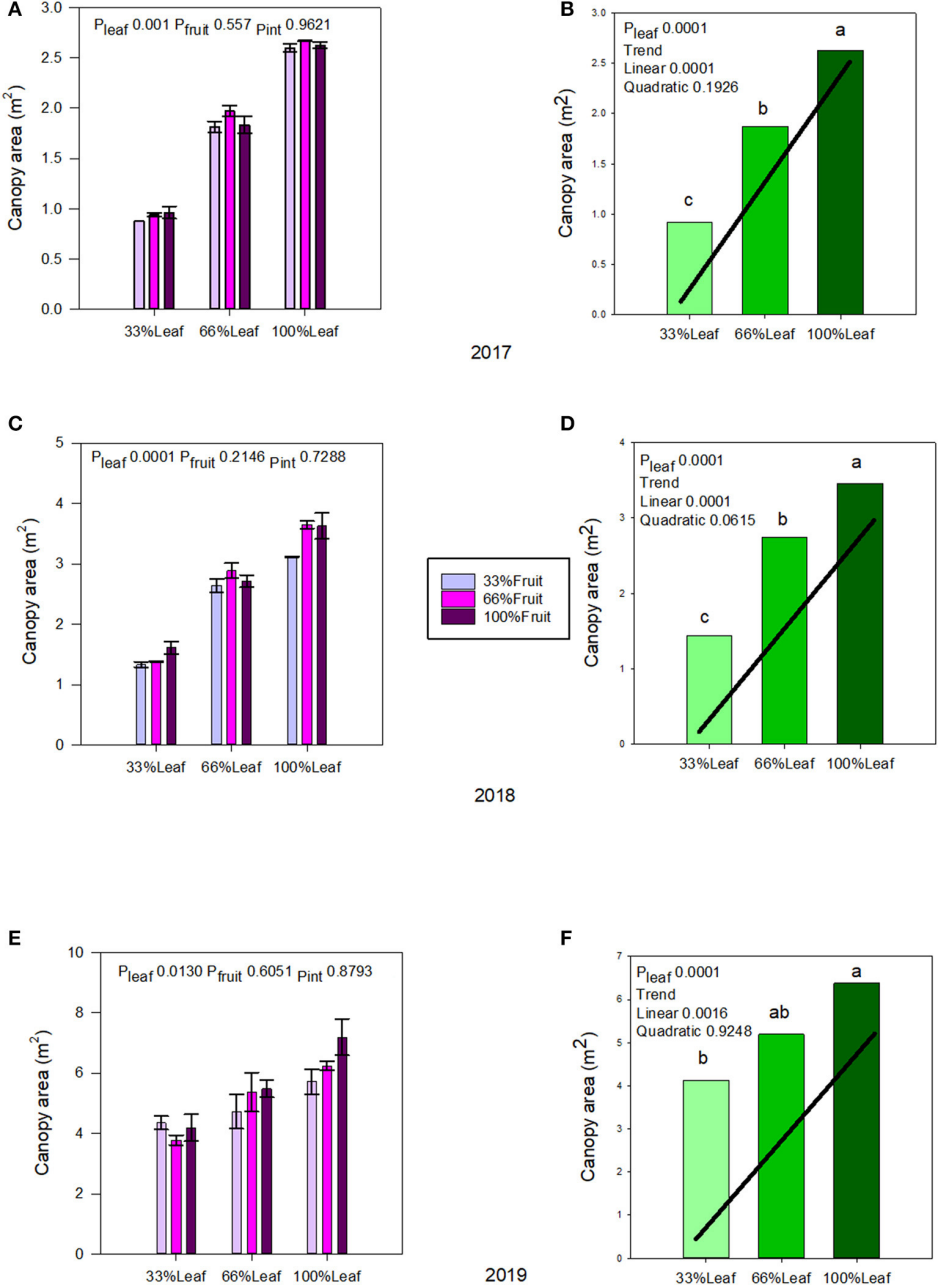
Effect of defoliation and fruit removal on the canopy area of Cabernet Sauvignon grapevine in 2017 **(A,B)**, 2018 **(C,D)**, and 2019 **(E,F)**. The interactive effects and simple means with standard error of the mean in each year are presented in **(A,C,E)**. The main effect of defoliation with significant trend line are presented in **(B,D,F)**. Columns with different letters in the main effect panel are significantly different at *P* < 0.05 according to Tukey's HSD.

Canopy area was affected by defoliation, but not with fruit removal or its interaction with defoliation during the experimental years (2017–2018) (Figures 2A,C). There was a strong linear trend, as expected with defoliation; where removing 66% of canopy area resulted in a 65% decrease of it in 2017 (Figure 2B); and a 58% in 2018 (Figure 2D). The carryover effects of source-sink adjustments on the canopy area in 2019 (Figures 2E,F) were evident. The strong linear decrease in 33%L continued to this year, as the grapevines did not recover from the removal of 66% of their leaf area, regardless of fruit removal in previous years.

**Figure 3:**
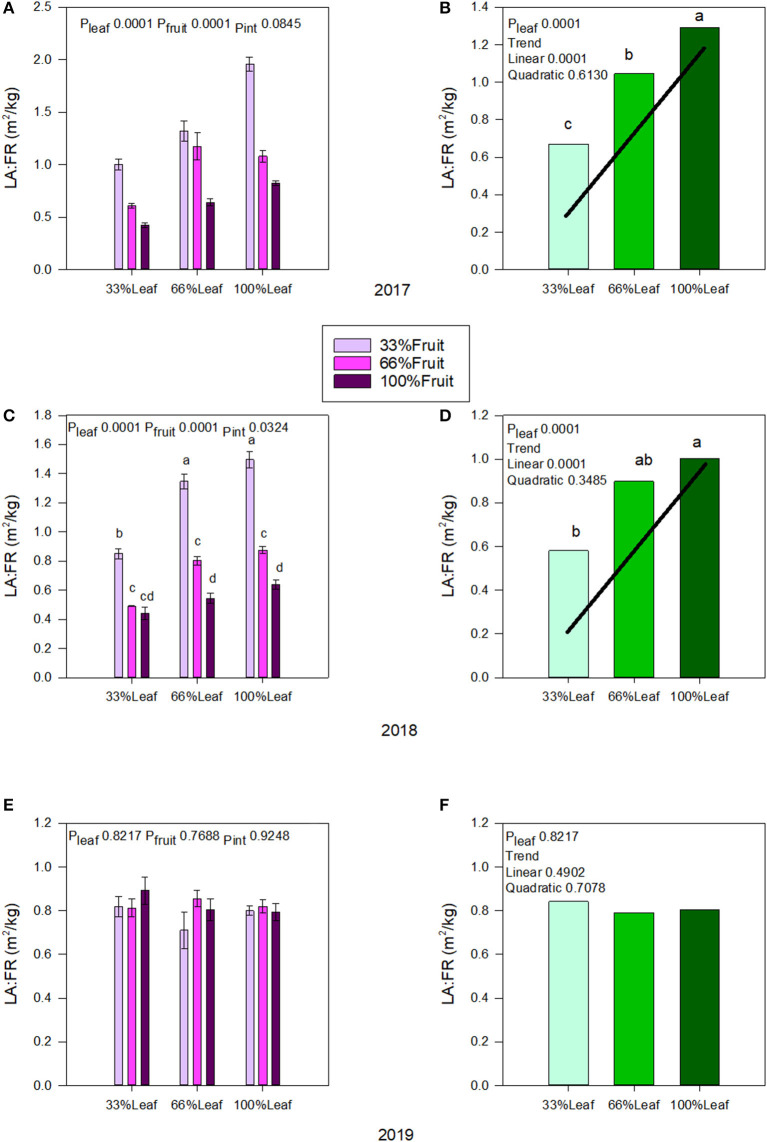
Effect of defoliation and fruit removal on leaf area to fruit mass (LA:FR) on Cabernet Sauvignon grapevine in 2017 **(A,B)**; 2018 **(C,D)**; 2019 **(E,F)**. The interactive effects and simple means with standard error of the mean in each year are presented in **(A,C,E)**. The main effect of defoliation with significant trend line are presented in **(B,D,F)**. Columns with different letters in main effect panels are significantly different at *P* < 0.05 according to Tukey's HSD.

In 2017, both defoliation and fruit removal affected leaf area to fruit ratio; however, the interaction amongst them was not significant (Figure 3A). There was a strong linear trend where leaf area to fruit ratio decreased by 47% when 66% of the canopy area was removed (Figure 3B). Conversely, we measured a strong linear trend where leaf area to fruit ratio increased by 56% this time when 66% of the fruit was removed. In 2018, defoliation and fruit removal interacted to affect the leaf area to fruit ratio (Figure 3C). Within the interaction, there was a linear trend where leaf area to fruit ratio decreased linearly as the number of clusters retained increased. We measured the greatest leaf area to fruit ratio with 100%L-33%F as well as in 66%L-33%F. The 33%L-100F and 66%L-100F had similar leaf area to fruit ratio (Figure 3D). There was no carry over effect of source-sink adjustments on leaf area to fruit ratio in 2019 (Figure 3E), nor was there an effect of main effects carrying over to this year (Figure 3F).

**Figure 4:**
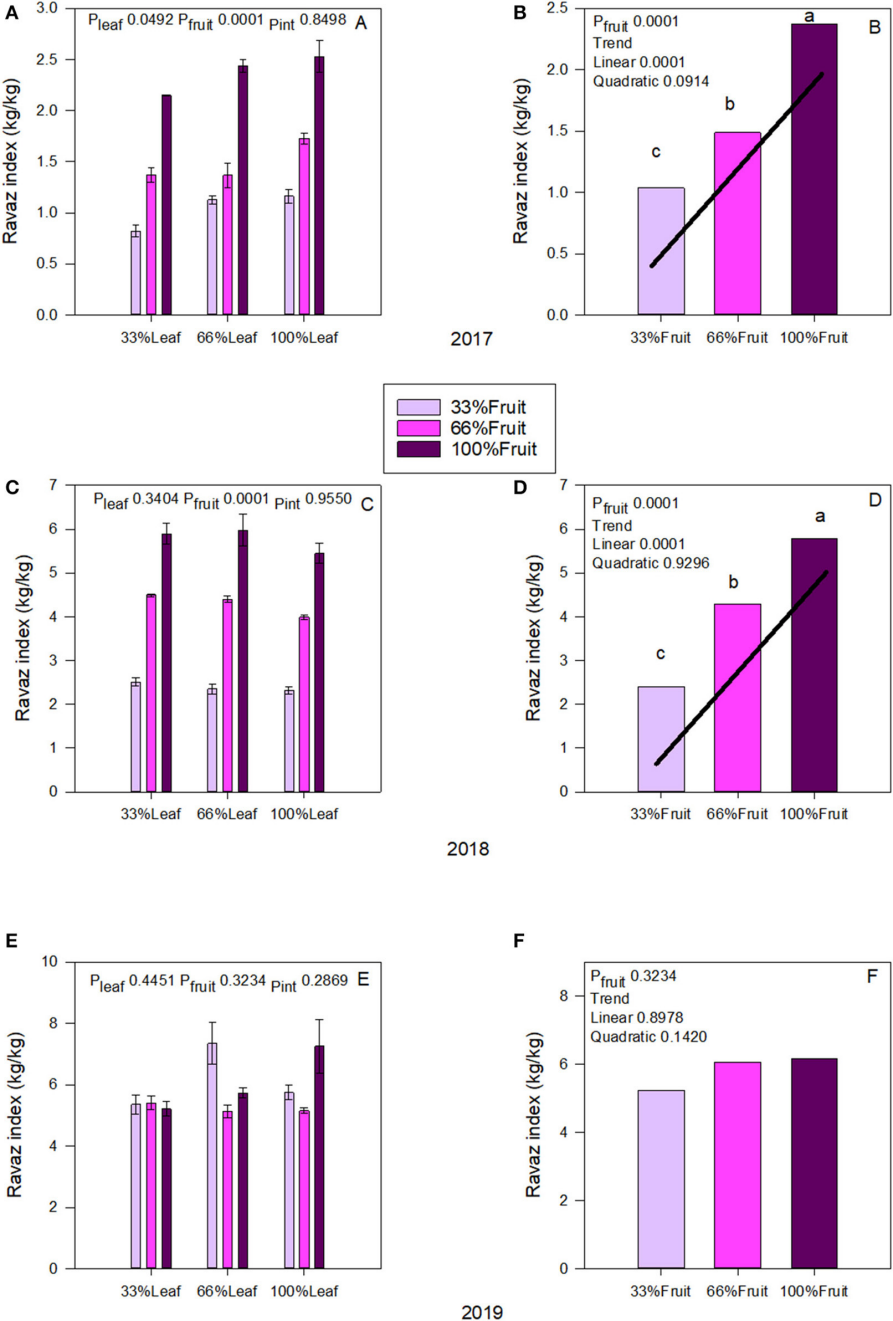
Effect of defoliation and fruit removal on yield and pruning mass (Ravaz Index) on Cabernet Sauvignon grapevine in 2017 **(A,B)**; 2018 **(C,D)**, and 2019 **(E,F)**. The interactive effects and simple means with standard error of the mean in each year are presented in **(A,C,E)**. The main effects of fruit removal with significant trend lines are presented in **(B,D,F)**. Columns with different letters in main effect panels are significantly different at *P* < 0.05 according to Tukey's HSD.

We measured an interactive effect of fruit removal and year on Ravaz Index (Figures 4A,C). During both experimental years, there was a strong trend of fruit removal on Ravaz Index (Figures 4B,D). In 2017, removing 66% of fruit resulted in a 56% decrease of Ravaz Index. We saw a similar response in 2018 as well. There was no effect of defoliation within the experimental years. We also did not measure an interactive effect of defoliation and fruit removal on Ravaz Index either. There was no carry over effect of source-sink adjustments on Ravaz Index in 2019 (Figure 4E); and we did not measure a carry-over effect of main effects of Ravaz index either (Figure 4F).

**Figure 5:**
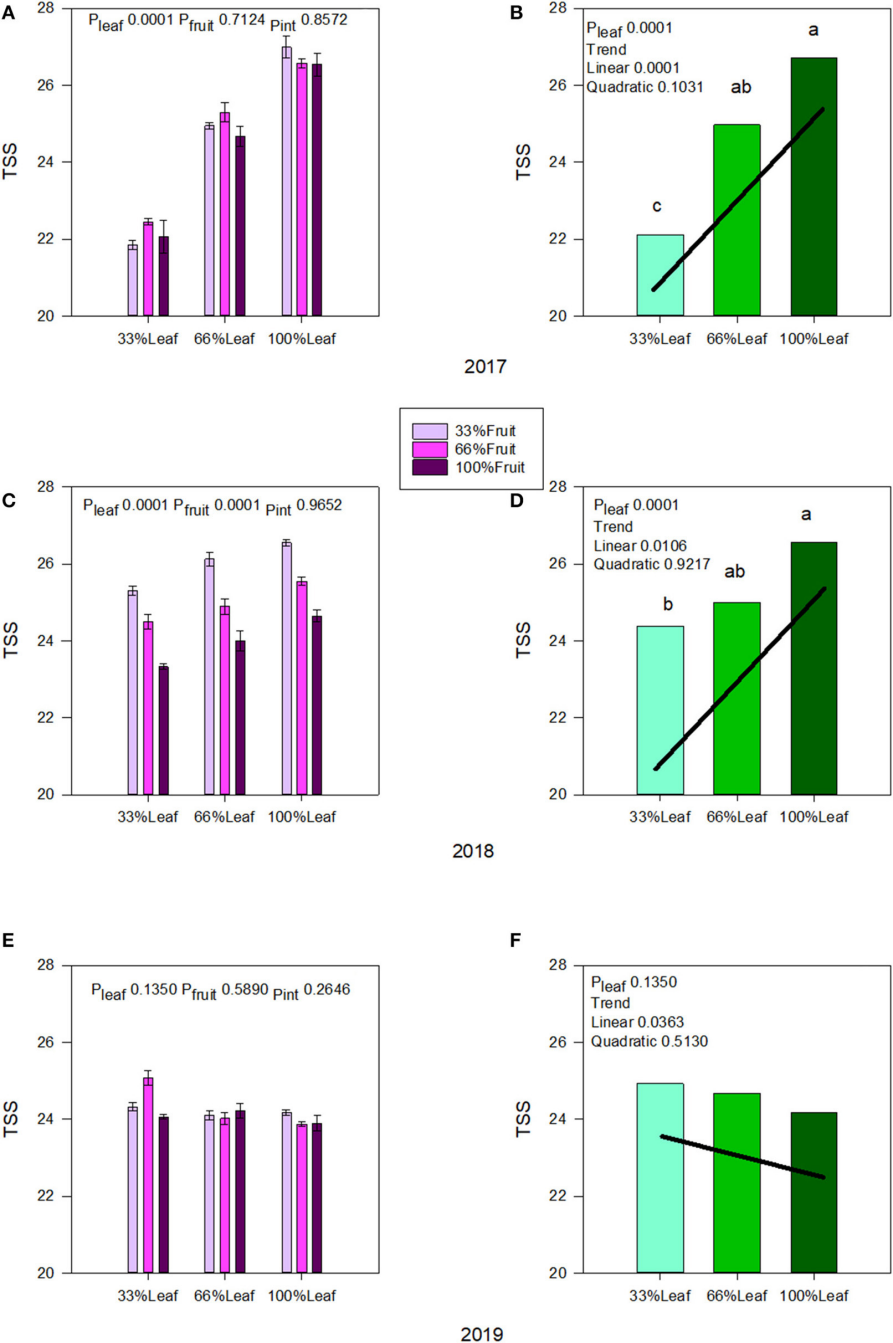
Effect of defoliation and fruit removal on berry total soluble solids of Cabernet Sauvignon in 2017 **(A,B)**; 2018 **(C,D)**; and 2019 **(E,F)**. The interactive effects and simple means with standard error of the mean in each year are presented in **(A,C,E)**. The main effects of defoliation with significant trend lines are presented in **(B,D,F)**. Columns with different letters in main effect panels are significantly different at *P* < 0.05 according to Tukey's HSD.

Berry TSS was affected strongly by the defoliation treatments during the experimental years, and we did not measure an interactive effect of defoliation and fruit removal in 2017 (Figure 5A) or in 2018 (Figure 5C). There was a linear increase in TSS as the severity of defoliation decreased in both experimental years (Figures 5B,D). The effect of fruit removal on speed of ripening in 2017 was negligible. However, we also saw a strong effect of fruit removal on berry TSS where it declined linearly with the decrease in fruit removal severity in 2018 (Figure 5C). This indicated a level of self-adjustment in previous grapevine season's applied treatments. Conversely, under free-growth in 2019 when the grapevine was allowed to sprawl without any defoliation and fruit removal, we saw a reversal of the trends with defoliation (Figures 5E,F). Although there were limited main and interactive effects, in the absence of fruit removal there was a linear decrease in TSS accumulation as the severity of defoliation treatments decreased, revealing the carry-over effect (Figure 5F).

### Effect of Source-Sink Adjustments on Leaf Gas Exchange Integrals

**Figure 6:**
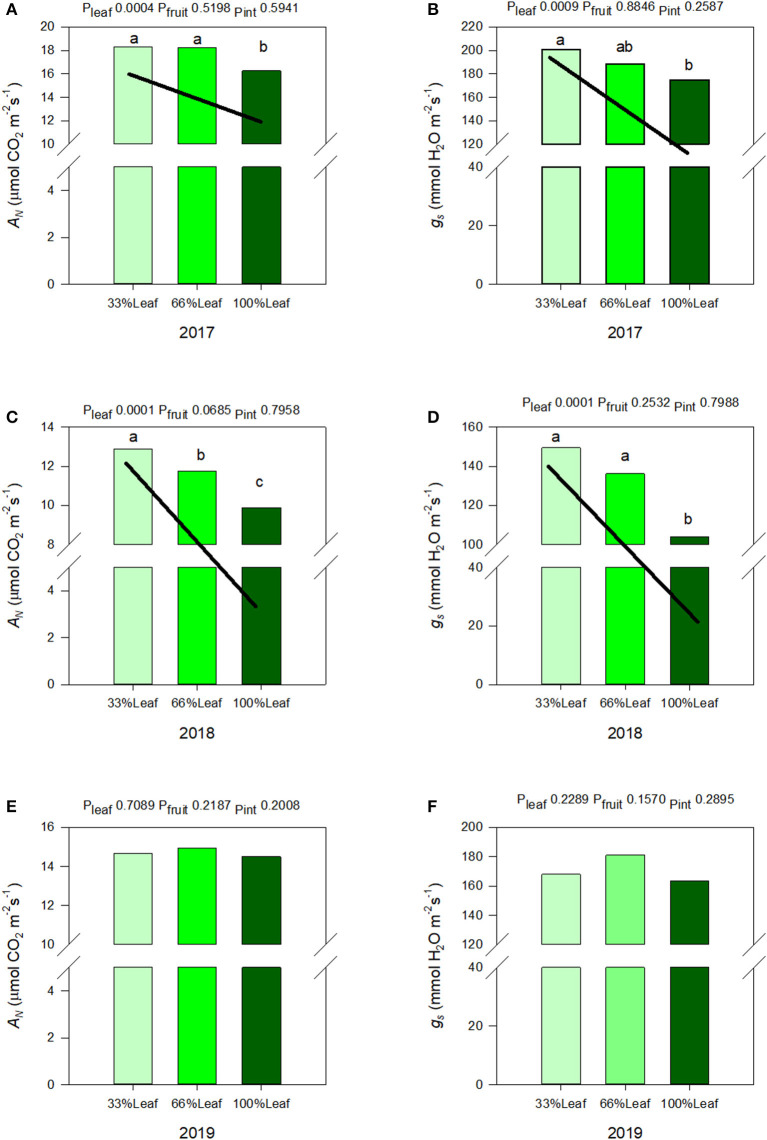
Effect of defoliation and fruit removal on integrals of net leaf carbon assimilation (*A*_*N*_) and stomatal conductance (*g*_*s*_) of Cabernet Sauvignon in 2017 **(A,B)**; 2018 **(C,D)**; and 2019 **(E,F)**. The interactive effects and simple means with standard error of the mean in each year are presented in **(A,C,E)**. The main effects of defoliation with significant trend lines are presented in **(B,D,F)**. Columns with different letters in main effect panels are significantly different at *P* < 0.05 according to Tukey's HSD.

We measured a significant year and defoliation interactive effect on *A*_*N*_, and *g*_*s*_ (Figure 6). There was never an interactive effect of fruit removal and year on any of the leaf gas exchange variables monitored. In regards to *A*_*N*_ during the experimental years (2017-2018), there was a strong linear trend where *A*_*N*_ and *g*_*s*_ increased linearly with the increase in the severity of defoliation (Figures 6A,B). In 2017, *A*_*N*_ and *g*_*s*_ increased by 10 and 13%, respectively, when 66% of leaves were defoliated. Defoliating 66% of leaves resulted in a 23% increase in *A*_*N*_, and a 30% increase in *g*_*s*_ integrals in 2018. In either experimental year we did not see an effect of cluster thinning on leaf gas exchange integrals or an interaction of defoliation and fruit removal.

The carryover effects to source sink adjustments in 2019 were inconsistent. We did not measure any significant effects of defoliation, fruit removal, or their interaction on *A*_*N*_, or *g*_*s*_ (Figures 6E,F). However, as mentioned above, the year effect on *A*_*N*_, and *g*_*s*_ were significant. There was a quadratic response to years where *A*_*N*_ and *g*_*s*_ declined from 2017 to 2018 but then increased significantly in 2019.

### Vine Mass, Starch, and Soluble Sugar Accumulation in Roots

**Figure 7:**
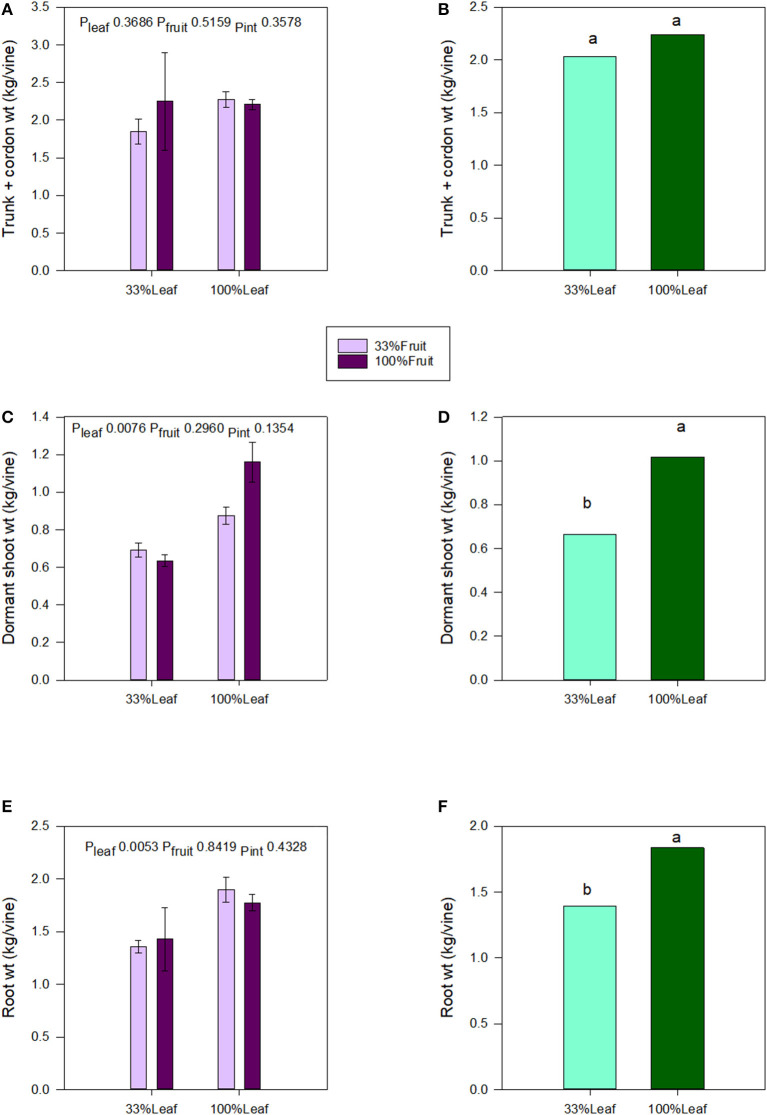
Effect of defoliation and fruit removal on trunk and cordon weight, dormant shoot weight, and root weight of Cabernet Sauvignon grapevine after 2 years of applied treatments. The interactive effects and simple means with standard error of the mean are presented in **(A,C,E)**. The main effect of defoliation is presented in **(B,D,F)**. Columns with different letters in main effect panels are significantly different at *P* < 0.05 according to Tukey's HSD.

We destructively harvested the grapevines following the 2018 growing season and separated them into roots, trunk, and aerial organs. Trunk and cordon masses were not affected by the defoliation, fruit removal, or their interactive effects (Figures 7A,B). Shoot and root masses were affected by the defoliation treatments. The shoot mass decreased by 1/3 with the 33%L treatment compared to the 100%L treatment (Figures 7C–E). Root mass decreased by 1/4 with the 33%L treatment when compared to 100%L treatment (Figure 7F). The total grapevine mass was also 20% lower in the 33%L treatment compared to 100%L treatment. Fruit removal did not affect plant biomass or biomass accumulation in plant organs, and there was no interaction of defoliation or fruit removal evident in our work.

**Figure 8:**
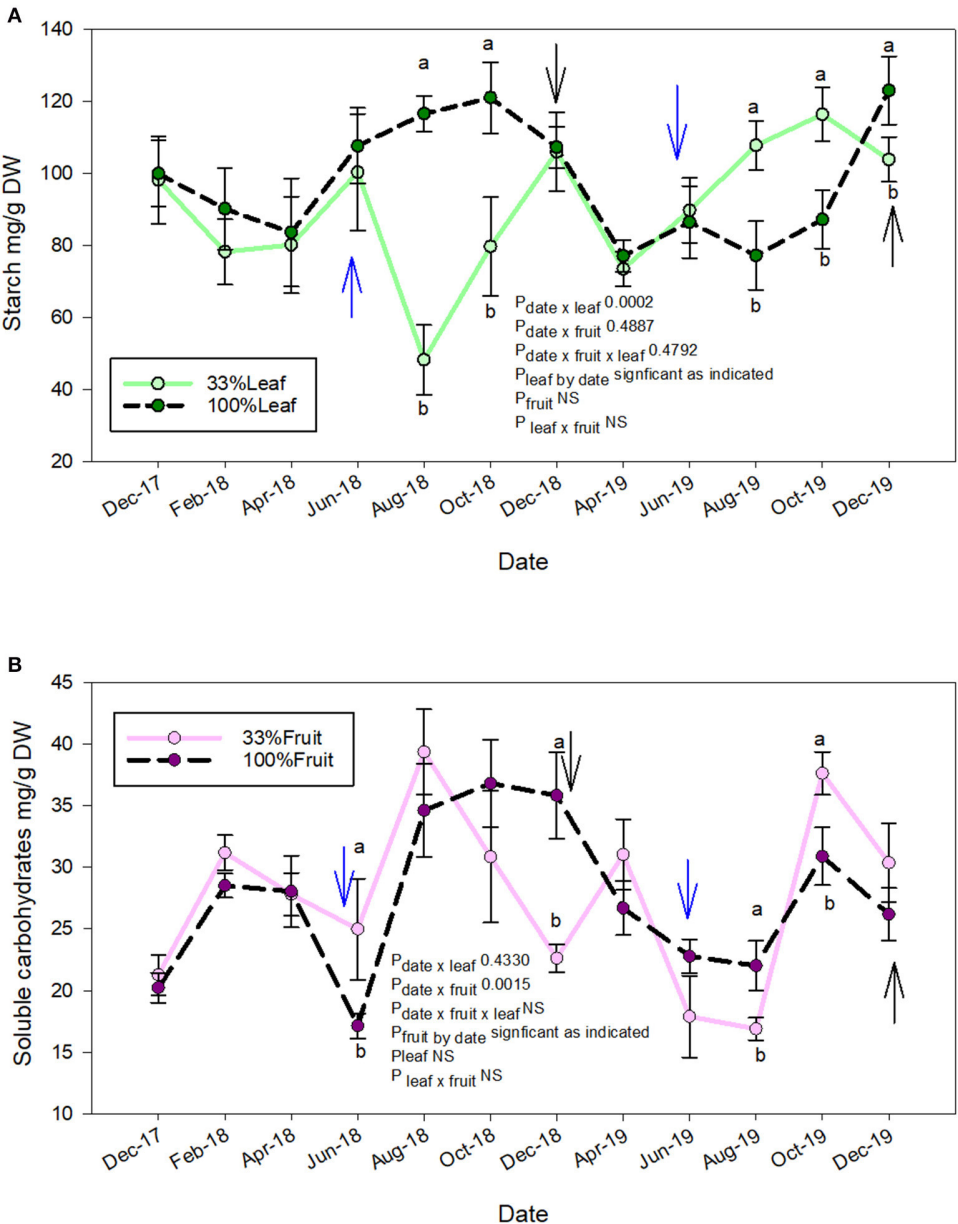
Repeated measures analysis of measurement date, defoliation and fruit removal on root starch **(A)** and total soluble carbohydrates **(B)**, respectively on Cabernet Sauvignon grapevine. Blue arrow = treatments applied. Black arrow: pruning. Bars on each measurement date indicate standard error of the mean. Dates with different letter indicate significant difference at *P* < 0.05 on said time according to Tukey's HSD.

The starch accumulation in grapevine roots was affected by defoliation treatments and time of sampling (Figure 8A). We did not measure an interactive effect of defoliation and fruit removal. The starch accumulation was affected at similar times during the experiment during each year. In 2018, the root starch content of 33%L was one-third of 100%L starting in mid-ripening until harvest (Figure 8A). The starch content of 33%L roots, however, equilibrated to the same content of 100%L by December 2018 when pruning was conducted. In 2019, when no treatments were applied to observe the carry over effects, we saw a reversal of this response. The starch content of root tissues of the 33%L treatments had *ca*. 20 and 40% more starch than 100%L in 2019 during mid-ripening through harvest.

Crop level rather than defoliation affected the soluble sugars content in the roots (Figure 8B). We did not measure the interactive effect of defoliation and fruit removal on soluble sugars at any time point during the experiment. In contrast to root starch accumulation (Figure 8A), the soluble sugars were greater in the 33%F (*ca*. 2×) when compared to 100%F at treatment application in 2018. However, their content decreased to $\frac{1}{2}$ of 100%F by December 2018 (pruning). In the follow-up year where grapevines grew without treatments, we saw transient differences in root soluble carbohydrates at mid-ripening where 100%F had greater content than 33%F. However, post-harvest in 2019, the soluble carbohydrate content in roots of 33%F was *ca*. 2× of 100%F prior to leaf fall. At pruning time, compared to the previous year; there was no difference.

### Phenology

**Figure 9:**
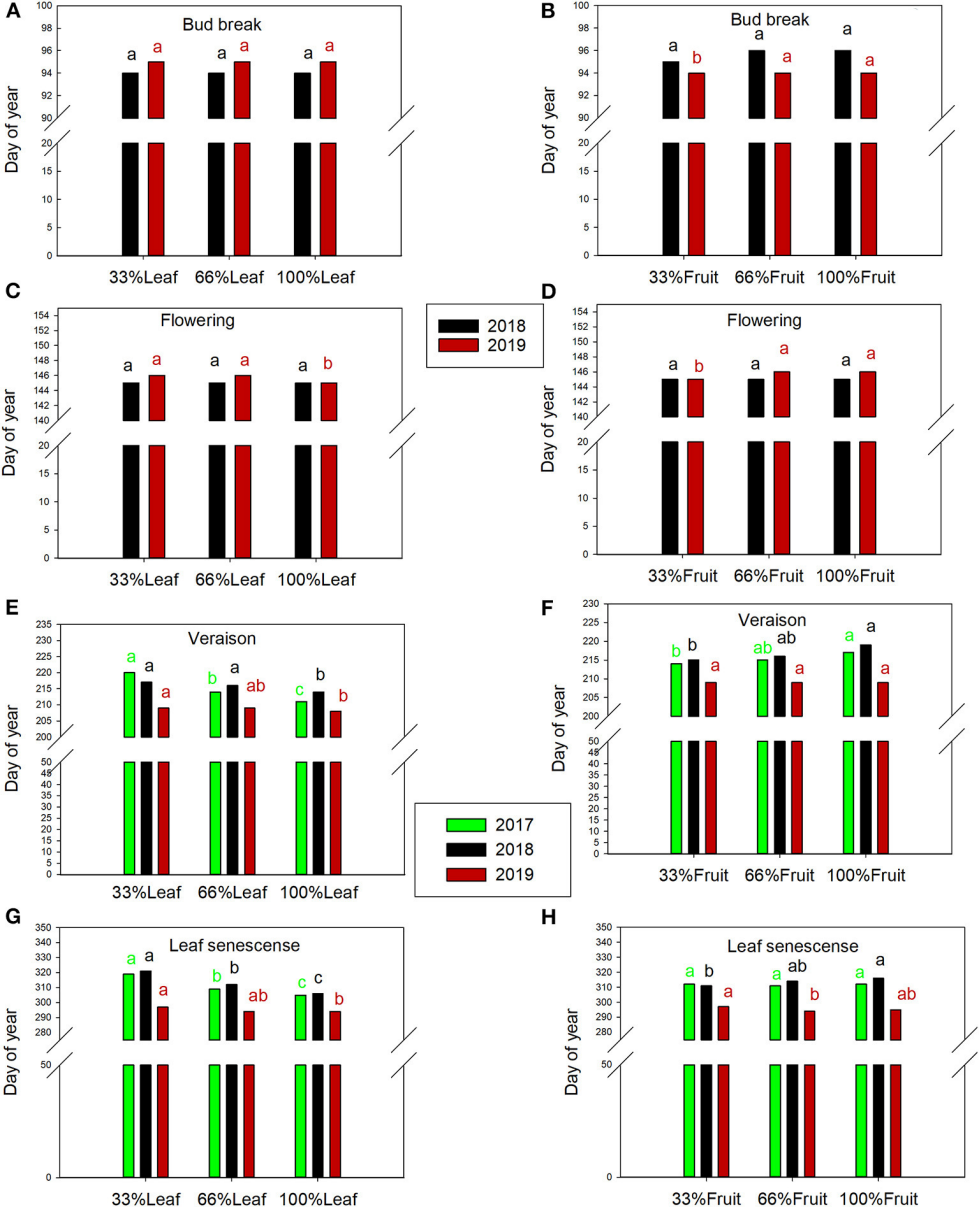
Effect of defoliation and fruit removal main effects on bud break **(A,B)**, flowering **(C,D)**, veraison **(E,F)** and leaf senescence **(G,H)** of Cabernet Sauvignon grapevine in 2017, 2018, and 2019 (green, black and red columns, respectively). Columns with different letters indicated by the same color are significantly different according to Tukey's HSD at *P* < 0.05. Interaction of defoliation and fruit removal not significant at either phenology measurement point in any year.

The day of bud break was not affected by defoliation treatments during the experimental years or the follow-up year of 2019 (Figure 9A). However, bud break was 1 day later in 2019 in the 66%F and 100%F treatments compared to 33%F (Figure 9B). The date of flowering was not affected by defoliation treatments in 2018 (Figure 9C). However, it was 1 day later with 100%L in 2019. Likewise, we did not see a shift in flowering date with fruit removal in 2018, but a carry-over effect was evident with 33%F being 2 days earlier than 66%F and 100%F in 2019 (Figure 9D).

In 2017, the treatments started having an impact few weeks after they were imposed. Veraison was delayed by defoliation treatments by *ca*. 5 days in 2017 and 2018 (Figures 9E,F). Leaf senescence was consistently delayed the defoliation treatments (Figures 9G,H). Extreme defoliation delayed leaf senescence by 9, 5, and 2 days in 2017, 2018, and 2019, respectively. Conversely, we saw a reversal of this trend where retaining more fruit delayed leaf senescence, albeit with a less strong effect than defoliation.

## Discussion

### Yield Components May Self-Compensate Amongst Them to Buffer Differences in Vine Balance

At the moment when the treatments were imposed, the proportions between the numbers of leaves to clusters were extremely different amongst treatments. These differences were self-balanced largely by harvest. Changes in berry size explained a great part of this variation. Typically, berry size is managed by limiting the access of grapevine to water (Torres et al., 2021). However, in this case, defoliated plants had a most certain lower consumption of water, and therefore, better water status (Abad et al., 2019), but this was concomitant to a reduction in berry mass which ruled out the water status as cause. Another common way to manipulate berry size is a delay in cluster thinning that encourages a competition of fruits early in their development, making berries smaller (Kok, 2011; Geller and Kaan Kurtural, 2013). Mechanistic experiments revealed that berry growth and the import of sugars may not occur in absence of the one or the other (Castellarin et al., 2011), and therefore, defoliation may have induced lower berry size through a reduction a sugar allocation (Torres et al., 2020). Berries per cluster were also affected by the severity of defoliation. This was a complex result as number of flowers, percentage of fruit set, or spontaneous fruit abortion may determine the number of berries at harvest development. However, in the 1st year of treatments, berry abortion after pepper-corn stage was evident. This was in fact the case, and vines in 33%L treatment displayed berry abortion close to veraison (Supplementary Figure 1) which was associated to carbon starvation (Domingos et al., 2016). Exogenous gibberellin applications are typically used in commercial table grape production to reduce cluster compactness through flower abscission (Lynn and Jensen, 1966). Endogenous gibberellin and auxin determine the number of berries set, therefore affecting the number of berries and thusly berry size (Grimplet et al., 2017). Although reductions in the number of berries per cluster were significant in the second season, berry abortion was not evident, which suggested this was an acclimation response to the treatments of the previous season. This was reported by previous studies where the number of inflorescence and flowers were reduced by defoliation during the previous season (Bennett et al., 2005).

### Ripening Is More Sensitive to Canopy Size Than Fruit Mass

Achieving vine balance has been remarked as key to achieve an adequate ripeness (Naor et al., 2002; Terry and Kurtural, 2011). However, balancing leaf area to fruit mass is a very precise task that involves a great amount of hand-labor (Kurtural et al., 2012, 2019) or investment in a large equipment park to conduct these practices mechanically (Geller and Kaan Kurtural, 2013). In this pursuit, trellis, vine spacing, canopy management, and irrigation have been proposed as indirect methods to optimize LA/FM (Hunter, 1998; Kliewer et al., 2000; Palliotti et al., 2011; Terry and Kurtural, 2011; Wessner and Kurtural, 2013; Torres et al., 2021). Thus, the concept of vine balance appears to work in a practical sense, as it is used to make cluster-thinning decisions and but not defoliation. Our data suggested that one unit of leaf area (or dormant pruning mass) was not equivalent to one unit of fruit mass. The main factor that made leaf area outweigh the value of fruit mass is that fruit is only a fraction of the total amount of carbon assimilated by leaves throughout the year is translocated to fruit (Vivin et al., 2001). Furthermore, leaves do not only assimilate carbon (Miller et al., 2000), and canopy size has deeper implications than fruit mass for root growth and the transduction of ripening signals. For instance, defoliation may suppress ABA signaling induced by water deficits (Ren et al., 2006), which would reduce berry ripening stimulation (Castellarin et al., 2007). In fact, ABA biosynthesis in leaves relies mostly on leaf induction rather than root signaling (Zhang et al., 2018). There is a feed-back mechanism between the production of auxin and cytokinin by leaves and roots, respectively; where auxins are biosynthesized by developing leaves and shoot tips and therefore stimulating root growth, and roots produce cytokinins that stimulate the growth of the aerial portions. Therefore, defoliation may not only reduce the amount of reduced carbon allocated to berries, but also to the ripening stimuli through endogenous plant growth regulators. Fruits are the fate of many growth regulators depending on the berry developmental stage (Conde et al., 2007), and thus, changes in crop load could potentially alter the balance between the amount stimuli and fates.

Therefore, leaf area to fruit mass is typically correlated to berry must soluble solids as in the 2nd year of study (Supplementary Figure 2) (Naor et al., 2002; Kliewer and Dokoozlian, 2005). Although, it is possible to find situations in which crop level (cluster thinning) does not compensate for defoliation as in the 2nd year of study (Parker et al., 2015). In fact, it is rather unusual to find lack of effects on ripening when two thirds of the clusters are removed from such an early stage as the present study. Finding only a significant effect of fruit removal in total soluble solids in the 2nd year could also suggest cumulative effects of crop level. It could be hypothesized that in the 1st year bearing 100% of the clusters, while not showing a reduction in soluble solids, may have taken a toll on plant reserves.

However, neither root mass nor starch content were impacted by the crop level in our work. Palliotti and Cartechini (1998) performed cluster thinning on three varieties over three seasons and found that cluster thinning did not affect must soluble solids. In years where rainfall was more abundant (Brillante et al., 2018), results of cluster thinning were compensated with larger berries (Wilson et al., 2014), with compensation of berry size similar to our results. Precisely, these kinds of results are those that disturbed the correlation between leaf area to fruit mass and berry total soluble solids (Supplementary Figure 2). This suggested that larger berries may offer higher resistance to increases in berry total soluble solids regardless of leaf area to fruit mass (Wessner and Kurtural, 2013). This hypothesis was supported by the response of grapevines submitted to water deficits that had smaller berries with higher soluble solids despite having much lower carbon assimilation rates (Brillante et al., 2017).

### Lower Leaf Area Rather Than Over Cropping Lead to Delayed Development

Development, which encompassed the timing of all physiological events recorded (phenology, ripening and cycles of carbohydrate storage), was delayed clearly by defoliation when treatments were in place, which excluded bud break and flowering. The initiation of each of these pheno-phases (bud break, flowering, veraison, ripe fruit, and leaf senescence) is quite complex as it may require more than one preexisting condition. For instance, the release from dormancy is often associated to the fulfillment of a chilling/thermal time accumulation requirement (Martínez-Lüscher et al., 2016), which supported the observation that all grapevines in the same site as this experiment would have a similar date of bud break. However, entering and exiting dormancy is also concomitant with major events of mobilization of soluble carbohydrates that condition the response of the latent bud (Rubio et al., 2014). Similarly, veraison may be modeled with thermal time (Parker et al., 2013) but ultimately requires a sucrose stimulus (Dai et al., 2014). Leaf senescence of deciduous plants is largely induced by shorter days and cooler temperatures, but as evidenced in our work, defoliation treatments delayed it. Other studies have suggested that leaves are able to sense source strength and delay leaf senescence accordingly (Miller et al., 2000). However, in our work, source strength was achieved through more leaves rather than better leaf net carbon assimilation performance. In both experimental years we witnessed the 33%L treatments assimilate more carbon compared to 100%L to compensate. However, it remains to be seen in future works if this is infact a carbon starvation effect or an artifact of plant water status. In fact, high sugar levels are one of the signals inducing natural leaf senescence (Moore et al., 2003; Parrott et al., 2005), and this can be modulated. Interestingly, this response was not conditioned by sink strength (crop level) or differences in leaf area in the final year, only by the practice of defoliation in our study.

### Successive Defoliations Depleted Plant Reserves, Inhibiting Root Growth, Ultimately Having a Carryover Effect on Grapevine Size and Yield

In the third year of study, no treatments were applied and therefore, all effects observed are attributable to cumulative effect of previous years' conditions. The so-called carryover effects have been discussed in relation to indirect observations, where the treatments were applied for several years (Martínez-Lüscher et al., 2017) or when historical series were analyzed (Lobell et al., 2007). In the case of defoliation, much direct evidence of carryover effects exists. For instance, Jermini et al. (2010) showed how defoliation caused by downy mildew induced severe reductions in the successive year's yield. Bennett et al. (2005) also reported severe reductions in yield, and these were attributed to reductions in clusters per vine, to reductions in berries per cluster, but not to changes in berry mass. In that study, there were changes in inflorescence per vine and flowers per inflorescence. Therefore, promoting root and canopy growth over the years has a strong cumulative effect on yields. Alternate bearing is an issue in some tree fruit crops such as mango, avocado, olive, pistachio, citrus, etc. [reviewed by Monselise and Goldschmidt (1982)] and fruit removal in those crops is not only performed aiming for in-season effects, but also to maintain consistent yield over the seasons. The carryover effects of crop level in grapevine have (not been reported to our knowledge) been reported less frequently than defoliation. Our results suggested that in fact grapevine is a perennial crop not very sensitive to alternate bearing.

Yield was associated with dormant season precipitation (Nelson et al., 2016; Martínez-Lüscher et al., 2017; Torres et al., 2021) or root and shoot starch content at budburst (Bennett et al., 2005; Torres et al., 2021). In our results, starch content of roots was only affected by defoliation in July and September samplings, which are coetaneous with sucrose stimulus to berry ripening. As root starch content fully recovered in all treatments, root mass was the only factor that would explain changes in yield in the successive season (Torres et al., 2021). In fact, in 2019, grapevines that were defoliated during the two previous seasons had lower root mass and fruit load as a carryover effect, which led to a faster recovery of starch reserves. Likewise, the carry over effects of defoliation were evident in leaf area, berries per cluster, and yield in the final year.

## Conclusions

After many efforts directed at balancing grapevine canopy by focusing on fruit removal, a renewed focus on maintaining an active leaf area with proper solar radiation exposure to clusters is needed. Our data indicated that carbon balance and translocation were more influenced by leaf area (source) rather than crop level (sink). The canopy leaf area and architecture determined the photosynthetic capacity which in turned initiated the sugar-induced growth. Once a large enough plant reduced carbon pool was available, berry enlargement and sugar allocation determined berry size. Canopy size also dictated how fast ripening progressed as well as storage of non-structural carbohydrates. Finally, we did not measure a direct physiological benefit of fruit removal in the primary metabolism of grapevine that could suggest one unit of leaf area would equate to one unit of fruit in the grapevine.

## Data Availability Statement

The raw data supporting the conclusions of this article will be made available by the authors, without undue reservation.

## Author Contributions

SK and JM-L conceived the trial. SK acquired the funding and analyzed the data. JM-L collected the data, executed the trial, and wrote the first version of the manuscript. Both authors contributed to the article and approved the submitted version.

## Conflict of Interest

The authors declare that the research was conducted in the absence of any commercial or financial relationships that could be construed as a potential conflict of interest.

## Publisher's Note

All claims expressed in this article are solely those of the authors and do not necessarily represent those of their affiliated organizations, or those of the publisher, the editors and the reviewers. Any product that may be evaluated in this article, or claim that may be made by its manufacturer, is not guaranteed or endorsed by the publisher.

## Supplementary Material

Supplementary Figure 1 Typical clusters observed only in 33%L treatments showing berry abortion induced by carbon starvation prior to veraison (31 August 2017). Kaolin clay residues are visible on the rachis.

Supplementary Figure 2 Relationship between yield to pruning weight (Ravaz index) and leaf area to fruit ratio (LA:FR) (A) in 2017, 2018, and 2019; and between LA:FR and berry total soluble soils in 2017 and 2018.
