# Supplementary figures and images for: Same Season and Carry-Over Effects of Source-Sink Adjustments on Grapevine Yields and Non-structural Carbohydrates
Source: Front Plant Sci. 2021 Jul 26;12:695319. doi: 10.3389/fpls.2021.695319 (PMC8350779; doi:10.3389/fpls.2021.695319)

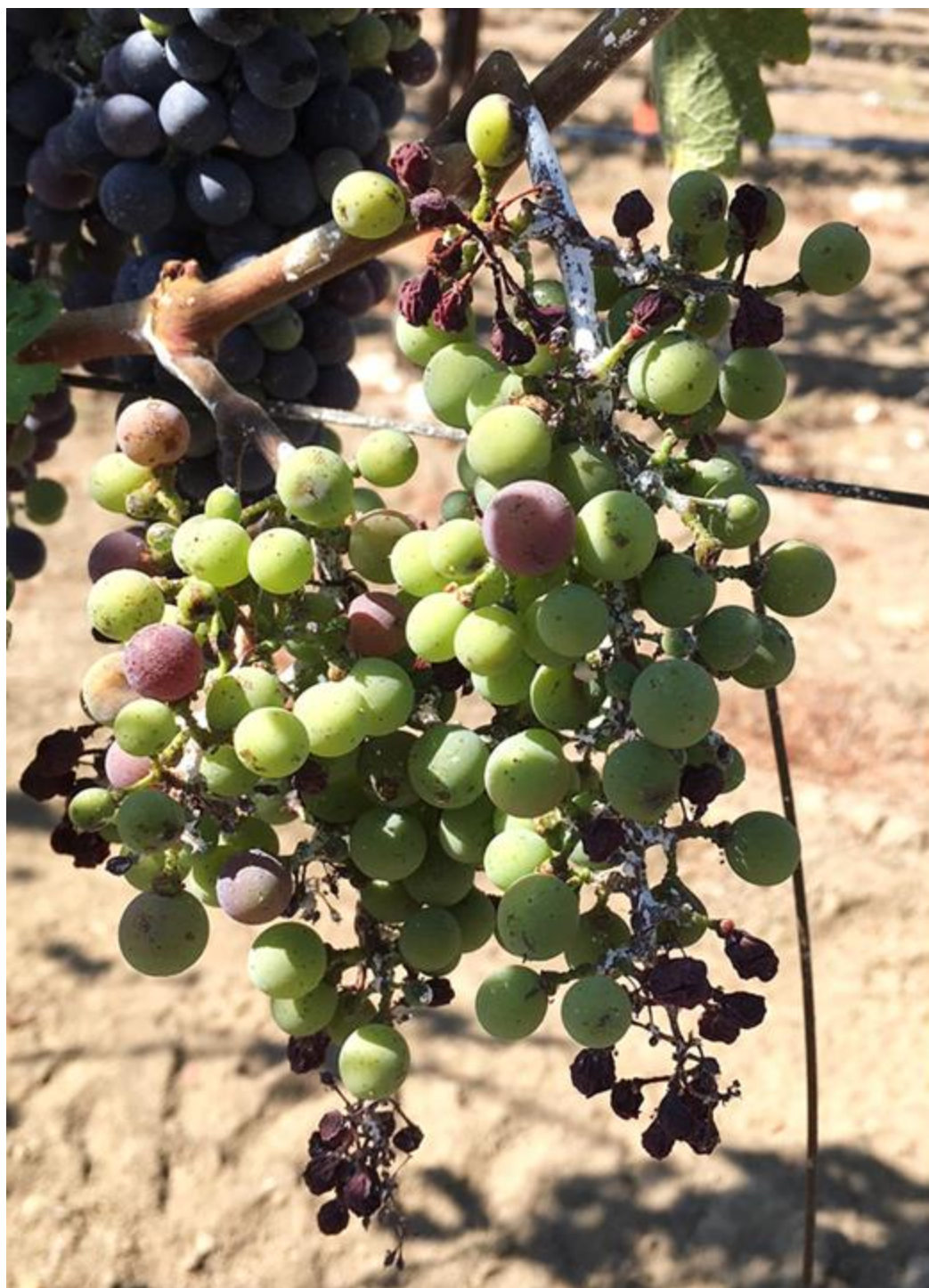

Figure S1

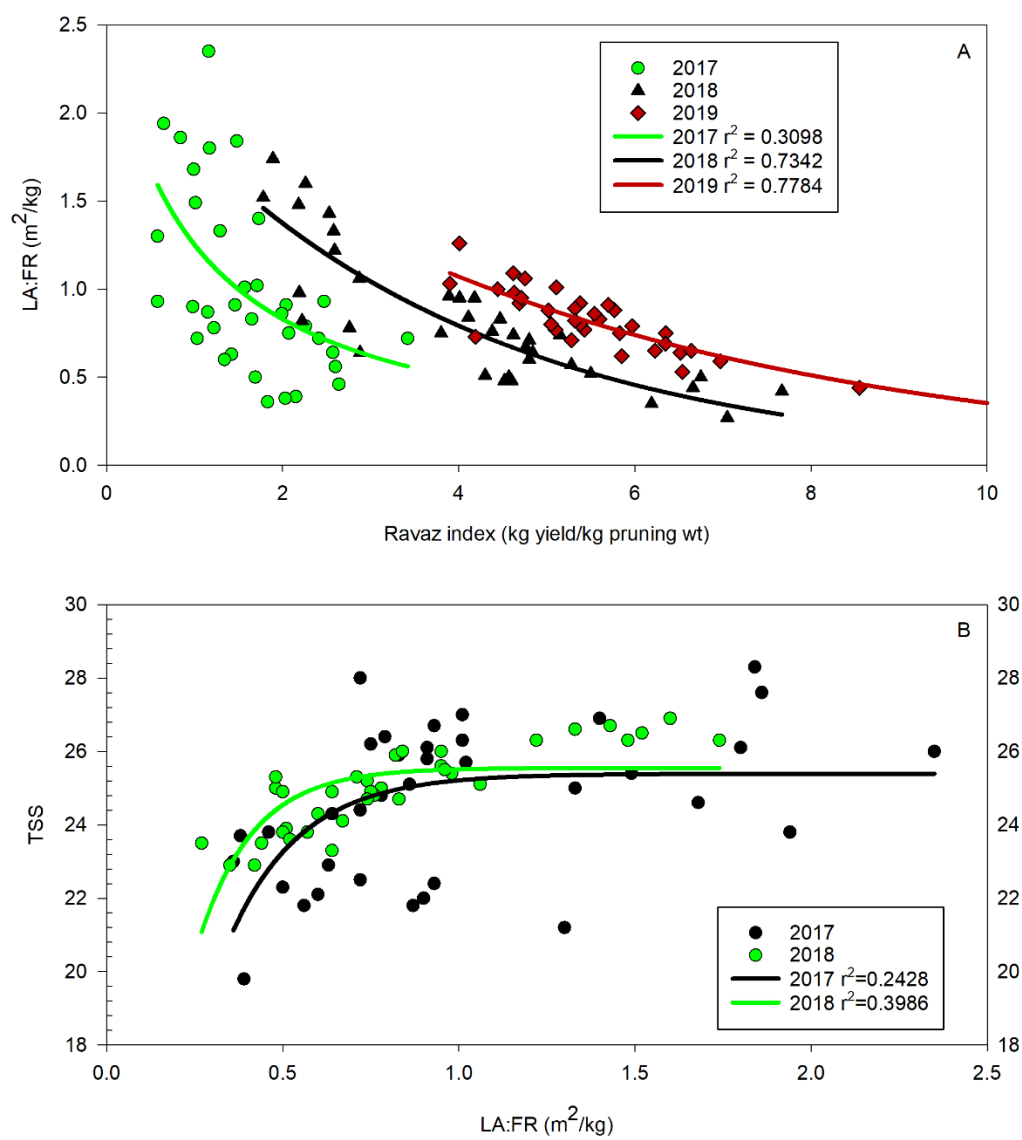

Figure S2.

Supplement: Supplementary Figure 1 — Typical clusters observed only in 33%L treatments showing berry abortion induced by carbon starvation prior to veraison (31 August 2017). Kaolin clay residues are visible on the rachis. [file Data_Sheet_1.PDF]
